# Hepcidin expression in the trigeminal ganglion and the oral mucosa in an oral ulcerative mucositis rat model

**DOI:** 10.1371/journal.pone.0284617

**Published:** 2023-04-20

**Authors:** Suzuro Hitomi, Tomotaka Nodai, Shoichiro Kokabu, Takemi Shikayama, Misa Sago-Ito, Chihiro Nakatomi, Kiyoshi Terawaki, Yuji Omiya, Masamichi Shinoda, Kentaro Ono

**Affiliations:** 1 Division of Physiology, Kyushu Dental University, Kitakyushu, Japan; 2 Department of Physiology, Nihon University School of Dentistry, Tokyo, Japan; 3 Division of Oral Reconstruction and Rehabilitation, Kyushu Dental University, Kitakyushu, Japan; 4 Division of Molecular Signaling and Biochemistry, Kyushu Dental University, Kitakyushu, Japan; 5 Division of Orofacial Functions and Orthodontics, Kyushu Dental University, Kitakyushu, Japan; 6 Kampo Research & Development Division, Tsumura Kampo Research Laboratories, Tsumura & Co., Ibaraki, Japan; 7 Kampo Research & Development Division, Research & Development Administration Department, Tsumura & Co., Ibaraki, Japan; Jagiellonian University Medical College, POLAND

## Abstract

Severe intraoral pain induces difficulty in eating and speaking, leading to a decline in the quality of life. However, the molecular mechanisms underlying intraoral pain remain unclear. Here, we investigated gene modulation in the trigeminal ganglion and intraoral pain-related behavior in a rat model of acetic acid-induced oral ulcerative mucositis. Oral ulceration was observed on day 2 after acetic acid treatment to the oral mucosa of male Wistar rats, causing spontaneous pain and mechanical allodynia. Deoxyribonucleic acid microarray analysis of trigeminal ganglion tissue indicated that *Hamp* (a hepcidin gene that regulates cellular iron transport) was the most upregulated gene. In the oral ulcerative mucositis model, the upregulation of *Hamp* was also induced in the ulcer region but not in the liver, with no increase in hepcidin levels in the plasma and saliva, indicating that hepcidin was produced locally in the ulcer region in the model. Systemic antibiotic pretreatment did not increase the mRNA levels of *Hamp* in the trigeminal ganglion and ulcer regions. Hepcidin injection into the oral mucosa enhanced neuronal excitability in response to noxious mechanical stimulation of the oral mucosa in trigeminal spinal subnucleus interpolaris/caudalis neurons. These results imply that oral ulcerative mucositis induces oral mucosal pain because of infectious inflammation of the ulcerative area and potentiates *Hamp*, which represents anti-bacterial and anti-peptidase gene expression in the ulcer region and trigeminal ganglion. The regulation of cellular iron transport by hepcidin is likely involved in oral ulcerative mucositis-induced pain.

## Introduction

Orofacial inflammation and trigeminal nerve injury cause intraoral pain because of the sensitization of nociceptive neurotransmission via plastic changes in the expression of several genes and proteins. Oral ulcerative mucositis (OUM) is the most common oral mucosal disease worldwide. In individuals with a history of recurrent aphthous ulcers, OUM is known to be initiated by mucosal injury [1] and can be iatrogenic in dental patients with orthodontic appliances and unsuitable dentures [2–4]. OUM induces severe pain triggered by physical contact during eating and speaking, sometimes occurring spontaneously, resulting in a low quality of life. In particular, OUM develops throughout the oral cavity in almost all head and neck cancer patients undergoing chemoradiotherapy. The severe pain caused by OUM is one of chemoradiotherapy’s most serious side effects, which sometimes either delays or interrupt therapy or both [5–7].

Many basic studies have been conducted using animal models to explore new therapies for patients suffering from OUM. Compared to OUM induction by radiation of the tongue or scratching of the buccal mucosa [8, 9], treatment with acetic acid in the oral mucosa of the mandibular alveolus is an advantageous method for initiating oral ulcers because it is technically easy and inexpensive. Hence, we established a system to assay oral mucosal pain in conscious rats with OUM and demonstrated that local swab treatment with the traditional Japanese herbal medicine hangeshashinto and steroid ointment, and systemic treatment with anti-bacterial/anti-inflammatory drugs inhibits OUM-induced spontaneous pain and mechanical hypersensitivity [10–12]. According to previous studies, the severity of oral mucositis peaks on day 2 after acetic acid treatment; this peak corresponds to the intensity of OUM-induced pain [13]. Furthermore, on the peak day, inflammatory cell infiltration, leukocytosis, bacterial loading, and production of proinflammatory cytokines and prostaglandin E_2_ (PGE_2_) are induced in the OUM region [14, 15], similar to other OUM caused by radiation or mechanical injury [9, 16]. However, whether these mediators contribute to the OUM-induced pain caused by acetic acid treatment of the oral mucosa has not been fully elucidated.

It may be helpful to profile the modulation of gene expression in the trigeminal ganglion (TG) using microarray analysis to understand the mechanisms of OUM-induced pain at the molecular level. Systematic meta-analyses of microarray studies have identified a subset of 79 genes commonly regulated in the dorsal root ganglion (DRG) and spinal dorsal horn in animal models of inflammatory/neuropathic pain [17]. In particular, the regulation of islet-derived 3 beta (*Reg3b*) and chemokine [C-C motif] ligand 2 (*Ccl2*) were found to be the most notably upregulated genes [17]. In the TG, as in the DRG, *Ccl2* is upregulated following subcutaneous injection of capsaicin, and the expression of several genes (e.g., *Serpinb1b* [serine proteinase inhibitor, clade B, member 1b]) has also been shown to be regulated in an orofacial inflammatory pain model [18].

In the present study, we investigated daily changes in the relationship between these mediators and pain-related behavior following acetic acid treatment to identify the putative pain-associated mediators that induce pain in acetic acid-induced OUM. We then performed DNA microarray assays using TGs collected from the OUM model and analyzed the results using bioinformatics. Finally, we investigated the expression of the most upregulated gene *Hamp* in the TG and mucosal regions of the OUM model. Additionally, we analyzed the effect of hepcidin injection in the oral mucosa on the neuronal response in spinal trigeminal subnucleus interpolaris/caudalis (Vi/Vc) neurons.

## Materials and methods

### Animals

Male Wistar rats (*Rattus norvegicus*) (n = 172, 150–250 g for the periodontitis model, 250–350 g for the OUM and nerve injury models; Kyudo, Saga, Japan; Clea-Japan, Tokyo, Japan; Japan SLC, Japan) were randomly selected for each experiment. The rats were housed in pairs in clear cages and were maintained on a light-dark cycle (L:D, 12 h/12 h) in a temperature- and humidity-controlled room (21–23°C and 40–60%, respectively) with free intake to water and food. All experiments were conducted following the National Institutes of Health Guide for the Care and Use of Experimental Animals and the guidelines of the International Association for the Study of Pain [19] and were approved by the Animal Experiment Committee of Kyushu Dental University, Nihon University School of Dentistry and the Laboratory Animal Committee of Tsumura & Co. Additionally, the Animal Experimentation Committee of Kyushu Dental University (approval nos. 14–026 and 19–009) and Nihon University approved all the experiments (approval no. AP20DEN002). The study was reported in accordance with the ARRIVE guidelines. All surgery was performed under sodium pentobarbital or a combination of three anesthetics (butorphanol, medetomidine and midazolam) anesthesia, and all efforts were made to minimize suffering. The sample numbers in the experiments were decided according to previous reports [10, 11, 14, 20]. Rats were euthanized with CO_2_ exposure when the body weight was dropped below the 20%.

### OUM model

Rats were induced with oral ulcers using the previously described methods [13]. Eight-week-old rats were anesthetized with sodium pentobarbital (50 mg/kg, intraperitoneally (i.p.); Kyoritsu Seiyaku, Tokyo, Japan) or a combination of three anesthetics (butorphanol, 2.5 mg/kg; Meiji Seika Pharmaceutical, Tokyo, Japan), medetomidine (0.375 mg/kg; Xenoac, Fukushima, Japan), and midazolam (2.0 mg/kg; Sand, Tokyo, Japan). A small filter paper (9 mm^2^, Whatman, Maidstone, UK) was soaked in 50% acetic acid diluted with water and placed in the labial fornix region of the inferior incisors for 30 s. Naive rats that received only i.p. anesthesia without any treatment were used as controls. According to our previous study [14], sulfamethoxazole (800 mg/L)- and trimethoprim (400 mg/L)-containing water was provided as drinking water for two days before acetic acid treatment to reduce the severity of the oral ulcers and the induced pain behavior.

### Histological assays in oral ulcer area

The rats were divided into three groups (naive rats, rats on day 1 after acetic acid treatment, and rats on day 2 after acetic acid treatment). Under deep anesthesia with pentobarbital (100 mg/kg, i.p.), the rats were perfused through the heart with saline. The labial fornix region of the inferior incisors was then removed and fixed in 4% paraformaldehyde in phosphate buffer (PB) for 24 h. Ten-micrometer-thick sagittal sections of the labial fornix region were stained with hematoxylin and eosin.

### Measurements of pain-related behavior

The behavioral observation was performed in a manner blind to the experimental conditions. The observation order was randomized daily.

The mouth rubbing by both forelimbs was measured for 10 min (between 16:00 and 18:00) to evaluate spontaneous intraoral pain. Before the measurements, all rats were acclimated for three days (10 min, once per day) in a plastic cage (30 × 30 × 30 cm) used for the observations.

We used the stable intraoral-opening method to evaluate mechanical hypersensitivity in the oral mucosa, in which the lower lip of the rat was pierced with a magnetized ring to expose the oral mucosa in the labial fornix region of the inferior incisors, as described previously [13]. First, the rats were anesthetized with pentobarbital (50 mg/kg, i.p.). A magnetized needle (equivalent to 22 gauge; Daiso Sangyo, Hiroshima, Japan) was bent into a square form and used to pierce the mentum skin below the rat’s lower lip. One week after piercing, the rats were trained for 10 min per day to stably protrude their perioral regions through a hole in a handmade restrainer and expose the oral mucosa. A neodymium magnet (3 mm in diameter; Waki, Osaka, Japan) with 4 g weight was attached to the pierce for giving a constant vertical pressure. When the rats backed up and flicked away, the magnet was released from the pierce without damaging the mental region. After the rats was acclimated to the experimental conditions (approximately 2–3 weeks after piercing), the head withdrawal threshold in response to mechanical stimulation of the oral mucosa was measured by applying 0.02–6 g von Frey filaments (North Coast Medical, Morgan Hill, CA, USA), and handmade filaments (0.2 and 0.3 g). Mechanical stimulation was applied to the acetic acid-treated mucosal regions. The head withdrawal threshold was defined as the minimum pressure required to evoke head withdrawal in at least 3 of the 5 tests performed during mechanical stimulation.

### Bacterial and biochemical assays of the oral ulcer area

Before and on days 1 and 2 after acetic acid treatment of the oral mucosa, the oral mucosal tissue from the labial fornix region of the inferior incisors, including the area with the most severe mucositis, was extracted from rats that were deeply anesthetized with pentobarbital (100 mg/kg, i.p.). All instruments and lower lip skin were sterilized with 70% ethanol before tissue extraction from each rat. The removed lip was trimmed to a block with a mucosal surface approximately 3 × 3 mm and 1–2 mm thick.

The extracted oral mucosal tissues were bisected with a surgical scalpel, placed into a pre-weighed 1.5 mL plastic tube filled with 0.5 mL of sterilized PB saline (PBS), and ultrasonicated for 30 s (38 kHz, US-2; SND, Nagano, Japan) to leach out oral bacteria. Fifty μL of each sample was plated in duplicate onto brain-heart infusion agar (Nissui, Tokyo, Japan) without exceeding 500 counts per 90-mm-diameter dish. Colonies were manually counted after the plates were incubated overnight at 37°C. Anaerobic incubation was performed in an airtight container with AnaeroPack-Anaero, an O_2_-absorbing CO_2_-generating agent (Mitsubishi Gas Chemical, Tokyo, Japan). The remaining samples were immediately stored at -80°C and melted immediately before subsequent biochemical assays were conducted (within one week of sample collection).

Tissues extracted from a separate group of rats were homogenized in PBS with 10 mM indomethacin (Wako, Osaka, Japan) and a protease inhibitor cocktail (Sigma-Aldrich, St Louis, MO, USA) to examine the production of inflammatory mediators in the oral mucosa. Following centrifugation, the supernatants were collected, and total protein concentrations were measured using a protein assay kit (Bio-Rad Laboratories, Hercules, CA, USA). The PGE_2_ concentration was measured in triplicate using the following ELISA kit: Prostaglandin E2 ELISA Kit (Cayman Chemical, Ann Arbor, MI, USA). The concentrations were normalized to the total protein concentration.

### Periodontitis and nerve injury models

#### 1 Periodontitis model

A periodontitis model rats were made using the following methods based on previous studies [21, 22]. Since four weeks has been needed to make periodontitis model, the body weight of the periodontitis model rats was light at the time of preparation compared with the OUM and nerve injury models. The rats were anesthetized with a mixture of medetomidine (0.375 mg/kg), midazolam (2 mg/kg,) and butorphanol (2.5 mg/kg) and were fixed on a stereotaxic frame on their backs. The right second maxillary molar was tied with a 4−0 silk ligature in the cervical area. The ligation was retied with a new ligature one week later and confirmed every week until four weeks after the first ligation under 2% isoflurane anesthesia. Four weeks after the first ligation, the TG was removed.

#### 2 Nerve injury model

Inferior alveolar nerve (IAN) transection (IAN-X) was performed [23]. Briefly, the rats were anesthetized with a mixture of medetomidine (0.375 mg/kg), midazolam (2 mg/kg), and butorphanol (2.5 mg/kg). A small incision was made on the surface of the left facial skin and the masseter muscle, and the tissue was dissected to expose the alveolar bone. After the bone covering the IAN was removed, the exposed IAN was lifted and tightly ligated at two points of the nerve trunk just above the angle of the mandible and 1 mm proximal to the angle of the mandibular bone. Then, the IAN was transected and placed back in the mandibular canal without any discernible gap between the cut nerve ends. Next, a sham operation was performed, which was identical except for the IAN transection. Two weeks after the surgery, the TG was removed.

### Anti-bacterial pretreatments (AB)

Sulfamethoxazole (Wako) and trimethoprim (MP Biomedicals, Illkirch, France) were dissolved in drinking water at 800 and 400 mg/mL, respectively, to suppress bacterial loading into the ulcer region [14]. AB was continued from 2 days before acetic acid treatment to the end of the experiment.

### Microarray and bioinformatics

The rats were anesthetized with sodium pentobarbital (100 mg/kg, i.p.) and aortically perfused with cold saline to examine the changes in gene expression in the TG after the development of OUM. Whole TGs were dissected bilaterally, immediately placed into a solution (RNAlater; Thermo Fisher Scientific), and frozen at -80°C. Microarray analysis was conducted at the Chemical Evaluation and Research Institute (CERI; Saitama, Japan, www.cerij.or.jp). After the tissue was homogenized, total RNA was isolated using a commercially available column-based kit (RNeasy Mini Column; Qiagen, Hilden, Germany). A small aliquot from each sample was subjected to a quality control test using an Agilent 2100 Bioanalyzer with an RNA 6000 Pico kit (Agilent Technologies, Massy, France) to confirm that the quality and quantity of the RNA in the sample were suitable for use in the microarray. According to the Agilent procedure (One-Color Microarray-Based Gene Expression Analysis, ver 6.5, May 2010), SurePrint G3 Rat GE microarray analysis was performed at CERI. Hybridization occurred for 17 h at 65°C, an Agilent DNA Microarray Scanner was used to perform scanning, and the resulting image data were digitized via feature extraction (Agilent, version 10.7.1.1). Control TGs from age-matched naive rats (n = 5) were compared with samples taken from OUM model rats on day 2 after acetic acid treatment (n = 5). All samples passed the quality control inspection based on examining the normalized intensity values, principal component analysis, and hybridization controls.

The online bioinformatics tool DAVID (https://david.ncifcrf.gov/home.jsp, ver. 6.7, August 28, 2015) was used to identify groups of genes with similar functions based on the controlled Gene Ontology (GO) vocabulary. The output was a series of functionally related gene clusters (termed gene groups) that were ranked by the enrichment score, a statistical measure of the overall biological significance of each gene group relative to that of the entire gene list.

### RNA isolation, reverse transcription, and quantitative real-time PCR (qRT-PCR) analysis

Under deep anesthesia with pentobarbital (100 mg/kg, i.p.) or a mixture of medetomidine (0.375 mg/kg), midazolam (2 mg/kg), and butorphanol (2.5 mg/kg), the bilateral TGs, liver, and submandibular gland were extracted in naive and acetic acid-induced OUM models on day 2 with and without AB. Additionally, bilateral TGs were also extracted from periodontitis and IAN-X models. The TGs were immediately transferred to RNAlater (Thermo Fisher Scientific) and frozen at -80°C. Furthermore, the TG was trimmed and divided into the third, first, and second branches. The tissues were homogenized, and total RNA was extracted using the RNeasy Mini Kit (Qiagen) and reverse-transcribed into cDNA using Superscript IV (Invitrogen) and random hexamers (Thermo Fisher Scientific). Five replicates of five rats were used for each group. Furthermore, a 1.5 μL or 0.5 μL cDNA aliquot was amplified in a QuantStudio5 (Thermo Fisher Scientific), according to the manufacturer’s recommendations using PowerUp TM SYBERTM Green Master Mix (Thermo Fisher Scientific). The following primers were used: *Hamp* (forward, 5’-GCTGCCTGTCTCCTGCTT-3’; reverse, 5’-AGCCGTAGTCTGTCTCGTCTG-3’), NM_053469; *Reg3b* (forward, 5’-CTGGATTGGACTCCATGACC-3’; reverse, 5’-CTCCACTCCCATCCACCTC-3’), NM_053289.1; *Serpina3n* (forward, 5’-CTGGCAGCTGGCTGGTAT-3’; reverse, 5’-CTGGGAAGGAGAGGAGAGC-3’), NM_031531; *IL-6* (forward, 5’-CCTGGAGTTTGTGAAGAACAACT-3’; reverse, 5’-GGAAGTTGGGGTAGGAAGGA-3’), NM_012589.2; and *β-actin* (forward, 5’-CCCGCGAGTACAACCTTCT-3’; reverse, 5’-CGTCATCCATGGCGAACT-3’), NM_031144.22. The assays were performed in duplicate. The *ΔΔ*CT was used to quantify relative expression [24]. The relative number of these genes in each sample was normalized to that of *β-actin*, and the ratios were calculated relative to the average value of the naive group.

### Quantification of hepcidin

Under deep anesthesia with a mixture of medetomidine (0.375 mg/kg), midazolam (2 mg/kg), and butorphanol (2.5 mg/kg), blood and saliva were collected from naive and acetic acid-induced OUM models. Blood was collected from the heart in tubes containing ethylenediaminetetraacetic acid. Saliva was collected 30 min after i.p. administration of pilocarpine (4 mmol/kg, Kanto Chemical, Tokyo, Japan). Following centrifugation, supernatants of both blood and saliva were collected, and total protein concentrations were measured using a BCA protein assay kit (Thermo Fisher Scientific). Additionally, hepcidin concentrations in the supernatants were measured using the following ELISA kit: Rat *HAMP*/Hepcidin ELISA kit (LS-F24076; LSBio, Seattle, WA, USA).

### Western blotting

The rats were deeply anesthetized and transcardially perfused with saline. The oral mucosal tissue was dissected and homogenized in RIPA buffer (Nacalai Tesque, Kyoto, Japan) supplemented with protease inhibitors (1:100; TaKaRa, Shiga, Japan) at 4°C. After centrifugation for 30 min at 13,000 × g and 4°C, the protein concentration of the supernatants was measured using a BCA protein assay kit (TaKaRa). Homogenized samples were mixed with Laemmli sample buffer solution (Bio-Rad, Hercules, CA, USA) and heat-denatured for 5 min. The 50-microgram of samples were electrophoresed on Mini-PROTEAN TGX^TM^ precast gels (4–20%, Bio-Rad) and transferred onto polyvinylidene difluoride membranes using a Trans-Blot Turbo (Trans-Blot Turbo Transfer pack; Bio-Rad). After rinsing with Tris-buffered saline containing 0.1% Tween-20 (TBST), the membrane was incubated with 5% Blocking One-P (Nacalai Tesque) in TBST and overnight at 4°C with rabbit anti-hepcidin polyclonal antibody (1:2000, AB30760, Abcam) and mouse anti-β-actin monoclonal antibody (1:2000, sc-69879, Santa Cruz Biotechnology, Dallas, TX, USA). The membrane was incubated at room temperature for 2 h with horseradish peroxidase (HRP)-conjugated goat anti-rabbit polyclonal antibody or HRP-conjugated rabbit anti-mouse polyclonal antibody (1:5000; Cat No. 111-035-003 and 315-035-003, respectively; Jackson ImmunoResearch, West Grove, PA, USA). The immunoreactive proteins were visualized using a Western Lightning ECL Pro (PerkinElmer, Waltham, MA, USA). The intensity of each band was analyzed using a ChemiDoc MP system (Bio-Rad) and normalized to that of β-actin. The bands were quantified using the ImageJ software.

### Immunohistochemistry

The innervated site was investigated in the spinal trigeminal nucleus of the oral mucosa in the labial fornix region of the inferior incisors. Under deep anesthesia with i.p. administration of pentobarbital (100 mg/kg), capsaicin (100 mM) was applied to the oral mucosa in the labial fornix region of the inferior incisors of naive rats using a swab soaked in the drug solution (20 μL) for 5 min. Naive and OUM rats were used to examine neuronal activity in the spinal trigeminal nucleus in OUM rats. Five minutes after the capsaicin injection, rats were perfused with 0.9% saline followed by 4% paraformaldehyde in 0.1 M phosphate buffer. The brainstem and cervical cord were removed and post-fixed overnight at 4°C. Tissues were transferred to 20% sucrose (w/v) in PBS for two days for cryoprotection. The tissue was sectioned at a thickness of 30 μm using a freezing microtome. After incubation with 0.3% H_2_O_2_ for 30 min and blocking with 3% normal goat serum in PBS for 1 h, the free-floating sections were incubated with rabbit anti-phospho-p44/42 MAPK (ERK1/2) polyclonal antibody (1:1000, Cell Signaling, Danvers, MA, USA) for 72 h at 4°C or rabbit anti-c-Fos polyclonal antibody (1:2000, Santa Cruz Biotechnology). Then, the sections were incubated with biotinylated goat anti-rabbit IgG (1:600; Vector Labs, Burlingame, CA, USA) for 2 h and with peroxidase-conjugated avidin-biotin complex (1:100; ABC, Vector Labs) for 1 h at room temperature. After washing with 0.05 M Tris buffer (TB), the sections were incubated in 0.035% 3,3-diaminobenzidine-tetra HCl (Sigma–Aldrich), 0.2% nickel ammonium sulfate, and 0.05% peroxide in 0.05 M TB. Sections were washed in PBS, serially mounted on mas-coated slides, dehydrated in alcohol, and coverslipped. The number of the phosphorylated extracellular signal-regulated kinase (pERK1/2)- and c-Fos-IR cells in Vi/Vc was counted from every fourth section, and the mean number of pERK1/2- and c-Fos-IR cells (three sections/rat) was obtained from each animal.

### Neuronal recordings in the spinal trigeminal nucleus

Neuronal recordings were performed in the Vi/Vc using extracellular recording procedures reported previously [25, 26]. Initially, under deep anesthesia induced by i.p. administration of a mixture of medetomidine (0.375 mg/kg), midazolam (2 mg/kg), and butorphanol (2.5 mg/kg), the trachea was intubated for artificial respiration. The left jugular vein for pancuronium bromide (1 mg/kg/h, i.v.) was cannulated. Rats’ head was rigidly mounted in a stereotaxic apparatus, and the posterior margin of the occipital bone was removed to expose the brainstem. After removing the dura mater, the brainstem was soaked in a pool of mineral oil in the surrounding skin flaps. After preparation, the rat was immobilized and artificially ventilated; expiratory end-tidal CO_2_ (3.5–5.5%) and body temperature were monitored and maintained. Deep anesthesia was maintained with isoflurane (0.5–1.5%) mixed with oxygen during neuronal recording. The neurons in the Vi/Vc were recorded using enamel-coated tungsten microelectrodes (impedance = 9-11MΩ, 1000 Hz; FHC, Bowdoin, ME, USA). Based on their responses to mechanical stimulation of the oral mucosa of the labial fornix region of the inferior incisors, the recorded neuron was characterized as a nociceptive-specific neuron or a wide dynamic range neuron, as previously described [25]. Background (BG) activity was recorded for 60 and 5 s before mechanical stimulation. Then, mechanical stimuli with von Frey filaments (4, 10, and 26 g) and a camel hairbrush were applied to the neuronal mechanoreceptive field (mRF) in the oral mucosa for 5 s at 30 s intervals. The mean firing frequency (spikes/s) during mechanical stimulation was calculated. After characterizing the neurons, hepcidin (1 μg/10 μL, Fujifilm Wako Chemicals) was injected into the mRF in the oral mucosa. Thirty seconds, 0.5 h, and 1 h after the injection, neuronal BG activity, and responses to mechanical stimulation were recorded. After the neuronal recording, the rats’ brainstem including Vi/Vc was removed for confirming the recording site. Then, rats were euthanized with CO_2_ exposure.

### Statistical analysis

During the microarray analysis, the text file of the raw data, which contained the results of the intensity calculations performed for each chip, was normalized using GeneSpring GX (version 12.0; Agilent) and output as Microsoft Excel files. The normalized data for each probe are presented as fold changes compared to the corresponding data for naive rats, and Welch’s *t*-test was used to detect significant changes. Data normality was confirmed using the Shapiro-Wilk test in the other data. The data are expressed as mean ± standard error (SEM), and *n* represents the number of rats. Friedman and Dunn’s post hoc tests were used to identify differences in the mechanical threshold. One-way analysis of variance (ANOVA) was used to compare daily changes in other parameters. Following these ANOVA tests, Dunnett’s post hoc tests were used to detect significant time points. Unpaired and paired *t*-tests were used to compare data between the two groups. Differences were considered statistically significant at *p <* 0.05. Statistical analyses were performed using the GraphPad Prism 8 software package (GraphPad Prism Software Inc., San Diego, CA, USA).

## Results

### Profiles of the OUM model

**Fig 1:**
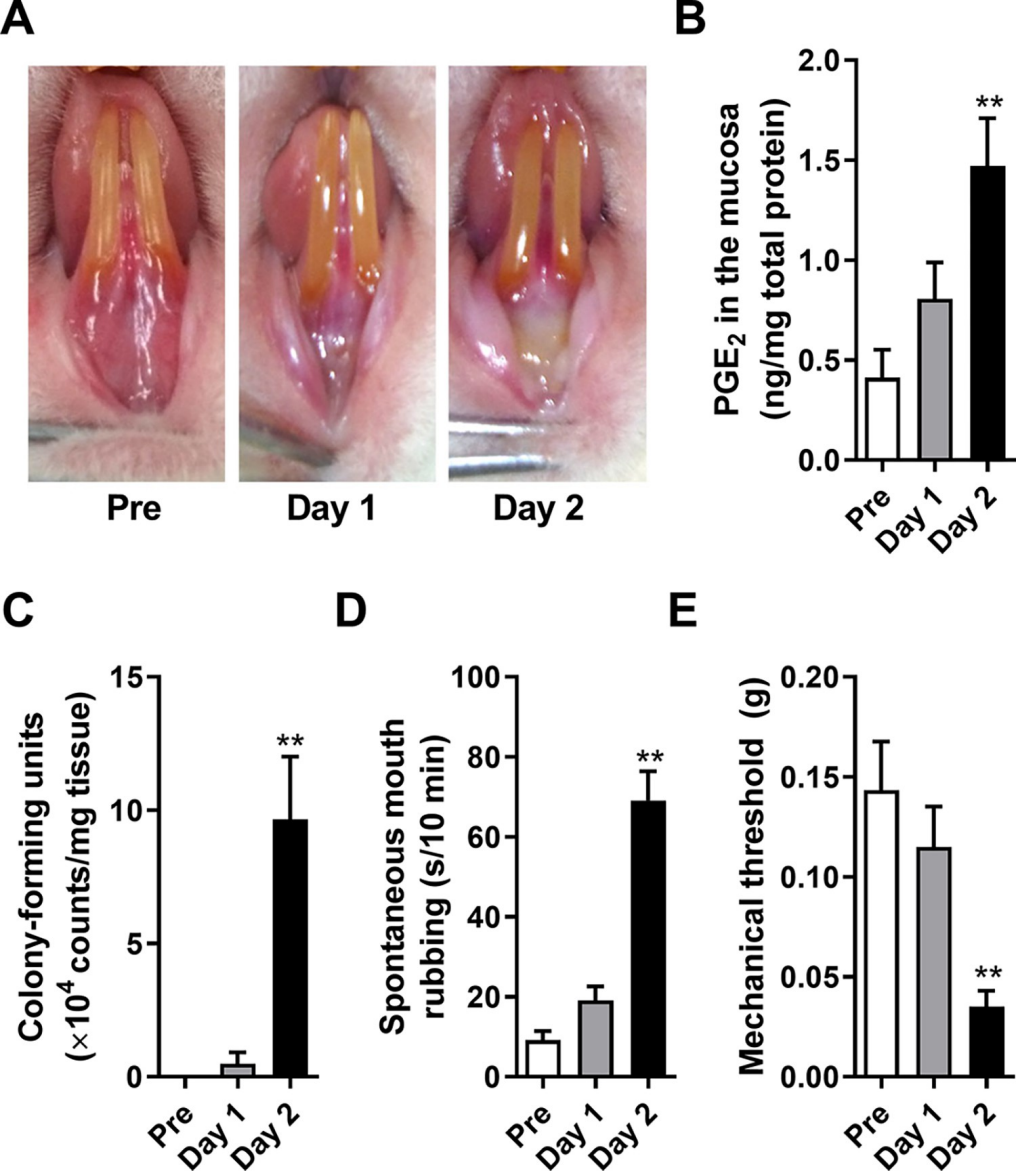
Development of oral ulcerative mucositis and pain induction following acetic acid treatment. **(A)** The visual appearance of the oral mucosa before the treatment (Pre) and on day 1 and day 2 after treatment with a 50% acetic acid-soaked filter paper (3 × 3 mm, 9 mm^2^) for 30 s. **(B)** Prostaglandin E_2_ (PGE_2_) production in the oral mucosa (n = 9 in each group). **(C)** Counts of colony-forming units (CFUs) under anaerobic conditions (n = 4 in each group). **(D)** Spontaneous mouth rubbing time for 10 min before (Pre) and on day 1 and day 2 after the acetic acid treatment (n = 5 in each group). **(E)** Head withdrawal threshold in response to the application of von Frey filaments to the oral mucosa before (Pre) and on day 1 and day 2 after the acetic acid treatment (n = 6 in each group). All bars represent the mean ± SEM. ** *p* < 0.01 compared with Pre, Dunnett’s post hoc test following one-way ANOVA (B, C and D) and Friedman and Dunn’s post hoc tests (E).

The acetic acid-treated area of the oral mucosa appeared slightly red on day 1 after treatment, although it was difficult to identify any differences from the healthy pretreatment mucosa (Fig 1A). However, the area demonstrated oral ulceration on day 2; occasionally, the dermis was exposed, and an ulcer developed. Compared with that in the pre-acetic acid-treated rats, the level of PGE_2_ in the oral mucosal tissues was significantly increased on day 2 (Fig 1B, *p* < 0.01 between Pre and Day 2). Under anaerobic conditions, the number of colony-forming units (CFUs) in PBS significantly increased on day 2 relative to that in the pre-rats (Fig 1C, *p* < 0.01 between Pre and Day 2). Compared to pretreatment, spontaneous mouth rubbing time and mechanical head withdrawal threshold significantly increased and decreased, respectively, on day 2 (Fig 1D, *p* < 0.01 between Pre and Day 2; Fig 1E, *p* < 0.01 between Pre and Day 2).

### Microarray analysis in the TG of the OUM model

To understand the modulation of neuronal gene expression in the OUM model, we conducted a large-scale pan-genome chip-based DNA microarray study using the Agilent SurePrint G3 Rat GE microarray and TG tissue samples. The data discussed in this study have been deposited in NCBI’s Gene Expression Omnibus (GEO) [27] and are accessible through the GEO Series accession number GSE83349. In microarray analysis, 14,823 genes were detected in all samples. First, we used arbitrary expression change cutoff values of more than 1.5-fold (upregulation) and less than 0.67 (downregulation), as recommended in the Affymetrix technical note [28], to identify significant differences in gene expression between naive and OUM model. The general evaluation criteria yielded 32 genes: 26 upregulated and six downregulated genes (Table 1). Of the 32 genes, *Reg3b*, *Csrp3*, *Arg1*, *RT1-Bb* and *C4a* matched the gene subset that has commonly been reported to be regulated across different pain models [17]. Functional clustering of the 32 significantly-regulated genes was performed based on GO annotations using the online bioinformatics tool DAVID [29]. This analysis of the enriched genes identified nine clusters of annotations within the GO “biological process” domain (Table 2). The top 3 clusters of annotations were “defense response,” “response to wounding,” and “inflammatory response,” as indicated by an enrichment score of 2.74.

**Table 1:** List of significant upregulated (>1.5 fold) and downregulated (<0.67 fold) genes.

**Upregulated genes**						
**#**	**Gene ID**	**Gene name**	**Protein name**	**Fold**	***P***	**Val**
1	84604	*Hamp*	Hepcidin anti-microbial peptide	3.65	0.007	2*
2	24618	*Reg3b*	Regenerating islet-derived 3 beta	3.33	0.011	2
3	24795	*Serpina3n*	Serine (or cysteine) peptidase inhibitor, clade A, member 3N	2.49	0.027	3
4	679566	*Fam150b*	Family with sequence similarity 150, member B	2.48	0.001	0
5	688047	*Lyc2*	Lysozyme C type 2	2.29	0.037	0
6	117505	*Csrp3*	Cysteine and glycine-rich protein 3 (cardiac LIM protein)	2.19	0.014	2
7	365871	*RGD1566380*	Hypothetical gene supported by NM_017187	2.09	0.020	0
8	113892	*Cml3*	Camello-like 3	1.89	0.021	0
9	25357	*Thrsp*	Thyroid hormone responsive	1.79	0.040	0
10	408239	*Mcpt1l4*	Mast cell protease 1-like 4	1.76	0.013	0
11	683761	*LOC683761*	Similar to RT1 class I, CE11	1.73	0.047	0
12	63840	*Per2*	Period circadian clock 2	1.70	0.002	0
13	29221	*Arg1*	Arginase 1	1.69	0.002	2
14	361787	*RT1-T24-1*	RT1 class I, locus T24, gene 1	1.68	0.014	0
15	29151	*Ucn*	Urocortin	1.67	0.017	0
16	314917	*Pkhd1l1*	Polycystic kidney and hepatic disease 1-like 1	1.67	0.030	0
17	303811	*Dvl3*	Dishevelled segment polarity protein 3	1.63	0.000	1
18	360533	*Rnf187*	Ring finger protein 187	1.62	0.038	0
19	24309	*Dbp*	D site of albumin promoter (albumin D-box) binding protein	1.62	0.000	0
20	309622	*RT1-Bb*	RT1 class II, locus Bb	1.59	0.004	1
21	24233	*C4a*	Complement component 4A (Rodgers blood group)	1.59	0.008	1
22	306657	*Irx2*	Iroquois homeobox 2	1.56	0.024	0
23	497913	*Gjc2 (CX47)*	Gap junction protein, gamma 2	1.56	0.039	0
24	100360872	*Mcpt1l1*	Mast cell protease 1-like 1	1.54	0.036	0
25	298031	*Susd1*	Sushi domain containing 1	1.52	0.000	0
26	499762	*Med22*	Mediator complex subunit 22	1.50	0.012	0
**Downregulated genes**						
**#**	**Gene ID**	**Gene name**	**Protein name**	**Fold**	***P***	**Val**
1	681186	*LOC681186*	Hypothetical protein LOC681186	0.66	0.030	0
2	29131	*Cartpt*	CART prepropeptide	0.65	0.013	1
3	114216	*S100a3*	S100 calcium binding protein A3	0.65	0.046	0
4	314262	*Plekhh1*	Pleckstrin homology domain containing, family H (with MyTH4 domain) member 1	0.62	0.026	0
5	316351	*Npas2*	Neuronal PAS domain protein 2	0.62	0.005	0
6	29237	*Penk*	Proenkephalin	0.62	0.006	3

Val, validation score: 0‒no evidence; 1‒weak, correlational evidence; 2‒strong, correlational evidence; 3‒causational evidence (see text for details).

*, Evidence published in the literature after our validation effort.

**Table 2:** Gene Ontology (GO) process in functional annotation clustering analyses of significant regulated genes.

E score	GO pathway name	Number of genes (list)	*P*
2.74	Defense response	7 (*Hamp*, *Reg3b*, *Serpina3n*, *Lyc2*, *Ucn*, *C4a*, *Penk*)	0.000
	Response to wounding	5 (*Reg3b*, *Serpina3n*, *Arg1*, *Ucn*, *C4a*)	0.008
	Inflammatory response	4 (*Reg3b*, *Serpina3n*, *Ucn*, *C4a*)	0.008
1.99	Regulation of system process	4 (*Csrp3*, *Ucn*, *C4a*, *Cartpt*)	0.022
1.42	Immune response	4 (*LOC683761*, *RT1-T24-1*, *RT1-Bb*, *C4a*)	0.042
	Antigen processing and presentation	3 (*LOC683761*, *RT1-T24-1*, *RT1-Bb*)	0.011
1.14	Behavior	4 (*Ucn*, *Cartpt*, *Npas2*, *Penk*)	0.045
	Neuropeptide signaling pathway	3 (*Ucn*, *Cartpt*, *Penk*)	0.008
0.76	Rhythmic process	4 (*Per2*, *Dbp*, *Cartpt*, *Npas2*)	0.003

GO pathway names are from the GO “biological process” domain. The enrichment (E) score is a measure of the significance of the gene group to the 32 significant regulated genes in Table 1.

Gene expression changes would be expected to occur mainly in the mandibular region of the TG than in the ophthalmic and maxillary regions of the TG because OUM was induced only in the inferior incisors’ labial fornix region. The general criteria for identifying significant changes in gene expression following oral ulcer development may be too strict because whole TG was used for the microarray analysis. Therefore, we applied a second set of arbitrary expression change cutoff values of more than 1.17-fold and less than 0.89-fold (*p* < 0.05) based on the assumption that gene expression would be regulated only in the mandibular region ([1.5 + 1 + 1]/3). The adjusted criteria yielded 495 genes with significant expression changes, including 287 upregulated and 208 downregulated genes. In addition to the five genes mentioned above, eight more significantly regulated genes were identified in the reported common gene set across pain models (S1 Fig). The expression of one of the most notably upregulated genes, *Ccl2*, increased by 1.12-fold (*p* = 0.111). The expression of the representative nociceptive channel genes *Trpv1* and *Trpa1* increased by 0.91 (*p* = 0.19)- and 0.91 (*p* = 0.29)-fold, respectively. These results are consistent with our previous study, which showed the absence of a significant difference in the expression of these genes between naive and OUM models using qPCR [15].

### Upregulation of *Hamp*, *Reg3b*, and *Serpina3n* in the TG of the OUM model

**Fig 2:**
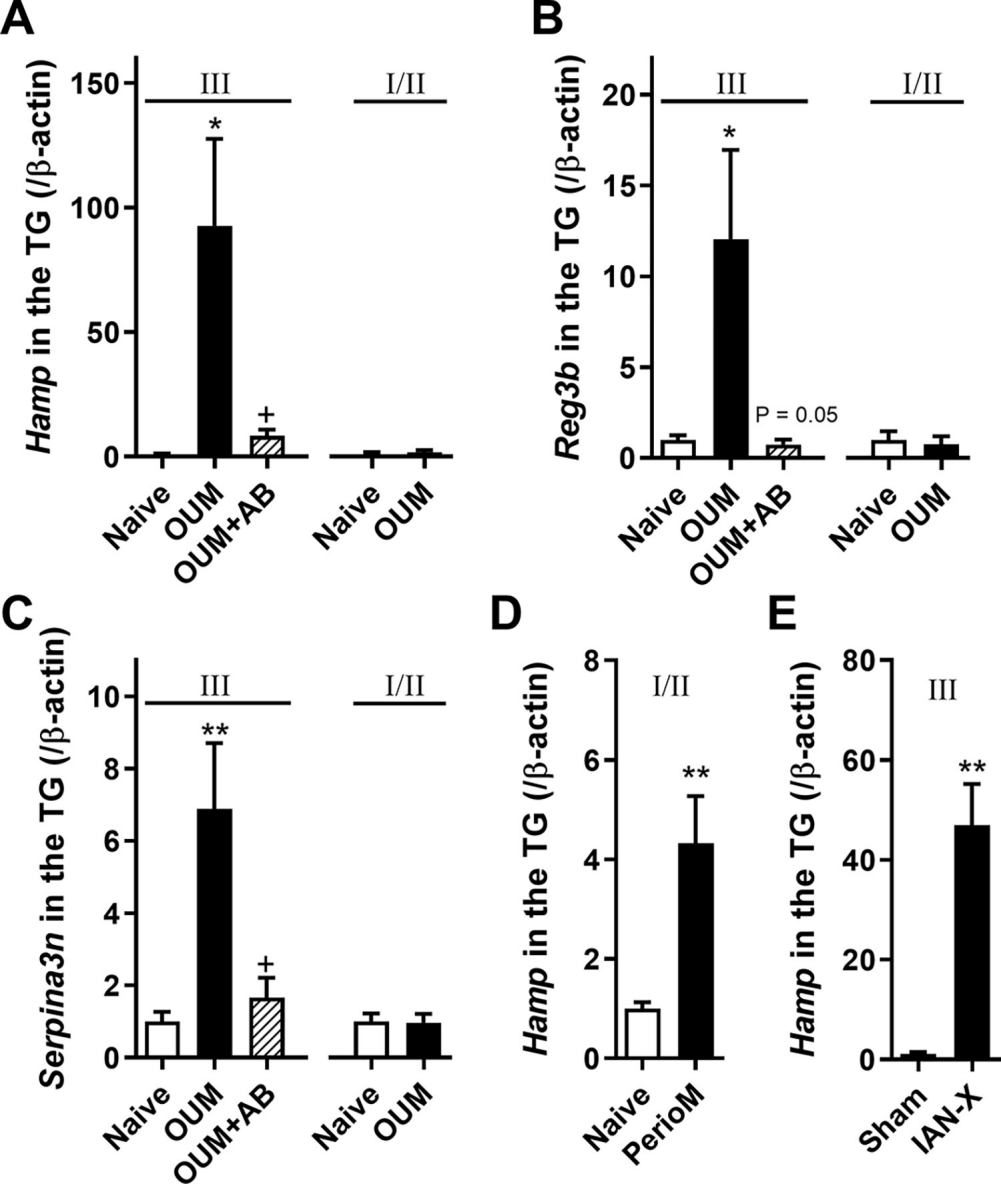
Changes in *Hamp*, *Reg3b* and *Serpina3n* mRNA levels in trigeminal ganglion (TG). All bars represent the mean ± SEM. **(A)** Relative expression of *Hamp* mRNA in the third (Ⅲ) and first/second (Ⅰ/Ⅱ) branches of TG on day 2 in Naive, acetic acid-treated oral ulcerative mucositis (OUM) and OUM with anti-bacterial pretreatment (AB) (n = 5 in each group). **p < 0.01 compared with Naive, Dunnett’s post hoc tests following one-way ANOVA. ^+^ *p* < 0.05 compared with OUM, Unpaired t-test. **(B, C)** Relative expression of *Reg3b* (B) and *Serpina3n* (C) mRNA in the third (Ⅲ) and first/second (Ⅰ/Ⅱ) branches of TG on day 2 in Naive, acetic acid-treated OUM and OUM with AB (n = 5 in each group). **p < 0.01 compared with Naive, Dunnett’s post hoc tests following one-way ANOVA. ^+^ *p* < 0.05, ^++^ *p* < 0.01 compared with OUM, Unpaired t-test. **(D)** Relative expression of *Hamp* mRNA in the Ⅰ/Ⅱ blanches of TG in the periodontitis model (perioM) rats (n = 4–5 in each group). ** *p* < 0.01 compared with Naive, Unpaired t-test. **(E)** Relative expression of *Hamp* mRNA in the Ⅲ blanch of TG in the inferior alveolar nerve transected (IAN-X) rats (n = 4 in each group). * *p* < 0.05 compared with Sham, Unpaired t-test.

Quantitatively measuring the mRNA levels of the top 1 upregulated gene *Hamp* on day 2 in the TG of naive and OUM model rats with and without AB was made using q-PCR to validate the modulation of gene expression in the model. Compared with naive rats, the mRNA level of *Hamp* in the third branch of the TG was significantly upregulated on day 2 in the OUM model and AB inhibited this increase (Fig 2A, *p* < 0.05 between Naive and OUM, *p* < 0.05 between OUM and OUM+AB). In the first and second branches of the TG, there was no difference in mRNA levels between naive and model rats (Fig 2A). The top two and three upregulated genes *Reg3b* and *Serpina3n*, respectively, were also significantly increased on day 2 in the third branch of TG in the OUM model compared with the naive group and AB inhibited this increase (Fig 2B, *p* < 0.05 between Naive and OUM, *p* = 0.057 between OUM and OUM+AB; Fig 2C, *p* < 0.01 between Naive and OUM, *p* < 0.05 between OUM and OUM+AB). The levels in the first and second branches of the TG did not change between naive and OUM rats (Fig 2B and 2C).

In the periodontitis model, the mRNA level of *Hamp* in the first and second branches of the TG was significantly increased compared to that in the naive group (Fig 2D, *p* < 0.01). That in the third branch of the TG was also significantly increased in the IAN transection group compared with the sham group (Fig 2E, *p* < 0.01). In summary, data from the meta-analysis of published pain microarray dataset (GDS2438) showed that L5 spinal nerve ligation increased the expression of *Hamp* in L5 DRG but not in L4 DRG compared with the sham operation (S2A Fig) (n = 4 in each group). In another meta-analysis of published pain microarray datasets (GDS2664) [30], the expression of *Reg3b* in the ipsilateral L5 DRG was significantly increased following L5 partial sciatic nerve ligation (PNL) and chronic constriction injury (CCI) compared with the sham operation (S2B Fig) (n = 4 in each group). Furthermore, expression of *Reg3b* and *Serpina3n* in DRG was significantly increased following spared nerve injury and spinal nerve ligation (SNL) and CCI models compared with each sham, respectively, in another previous meta-analysis dataset (GDS4625) [31] (S2C and S2D Fig).

### Expression of *Hamp* and hepcidin in the ulcer region in the OUM model

**Fig 3:**
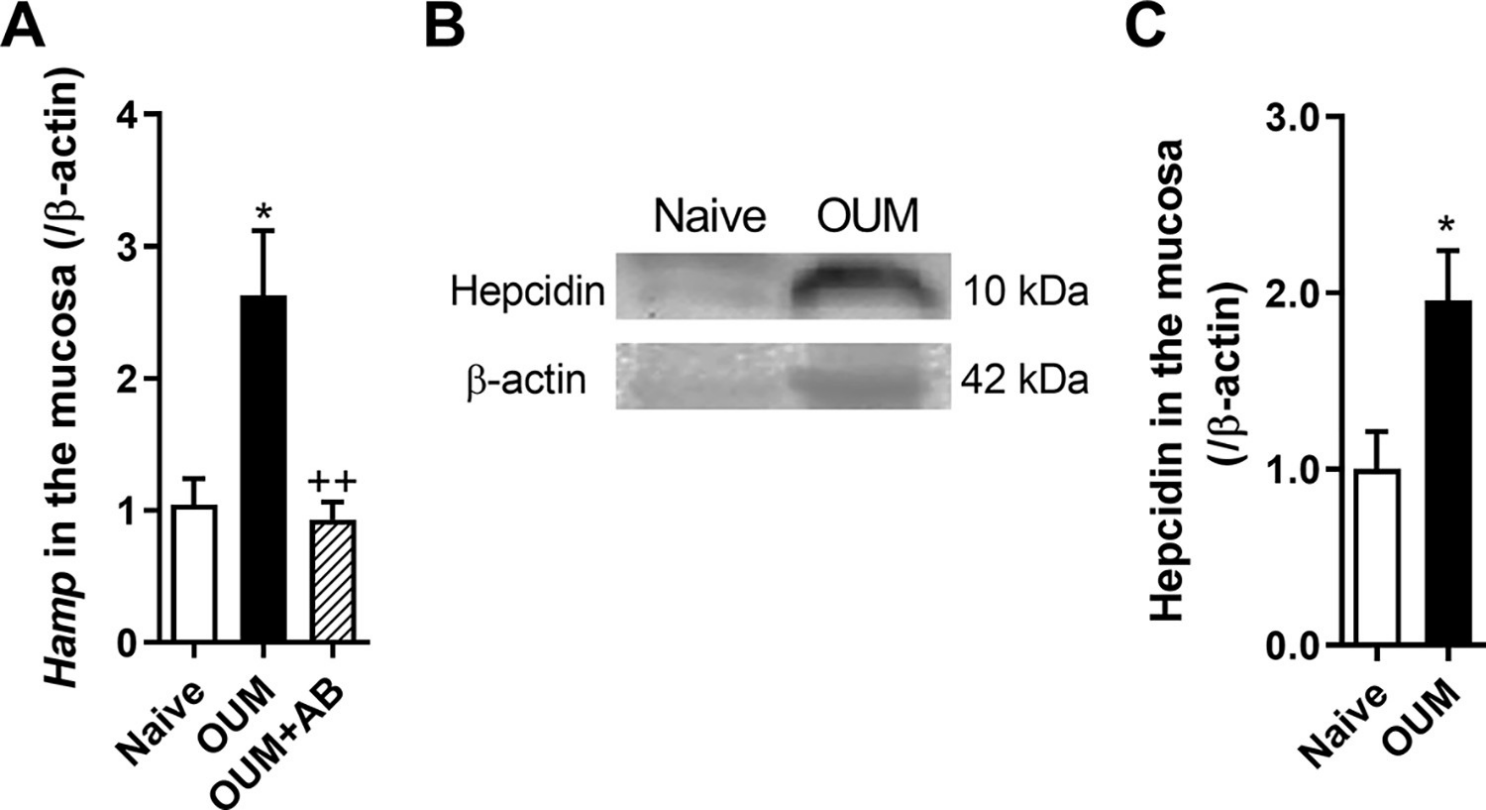
Changes in *Hamp* mRNA and hepcidin levels in oral mucosa in OUM model. All bars represent the mean ± SEM. **(A)** Relative expression of *Hamp* mRNA in the oral mucosa on day 2 in naive, acetic acid-treated oral ulcerative mucositis (OUM) and OUM with anti-bacterial pretreatment (AB) (n = 5 in each group). * *p* < 0.05 compared with naive Dunnett’s post hoc tests following one-way ANOVA. ^++^ *p* < 0.01 compared with OUM, Unpaired t-test. **(B)** Hepcidin in the oral mucosa on day 2 in naive and OUM. **(C)** The relative amount of hepcidin in the oral mucosa (n = 4 in each group). * *p* < 0.05 compared with naive. Unpaired *t*-test.

To examine whether *Hamp* mRNA is upregulated in the TG in a limited manner, mRNA levels in the ulcerative region were measured. *Hamp* mRNA levels in the ulcerative region were significantly higher in the OUM model group than in the naive group (Fig 3A, *p* < 0.05 between Naive and OUM, *p* < 0.01 between OUM and OUM+AB). AB inhibited this increase. Additionally, the amount of hepcidin was significantly increased in the ulcer region of the OUM model compared with that in the naive group (Fig 3B and 3C, *p* < 0.05).

### *IL-6*, *Hamp*, and hepcidin levels

**Fig 4:**
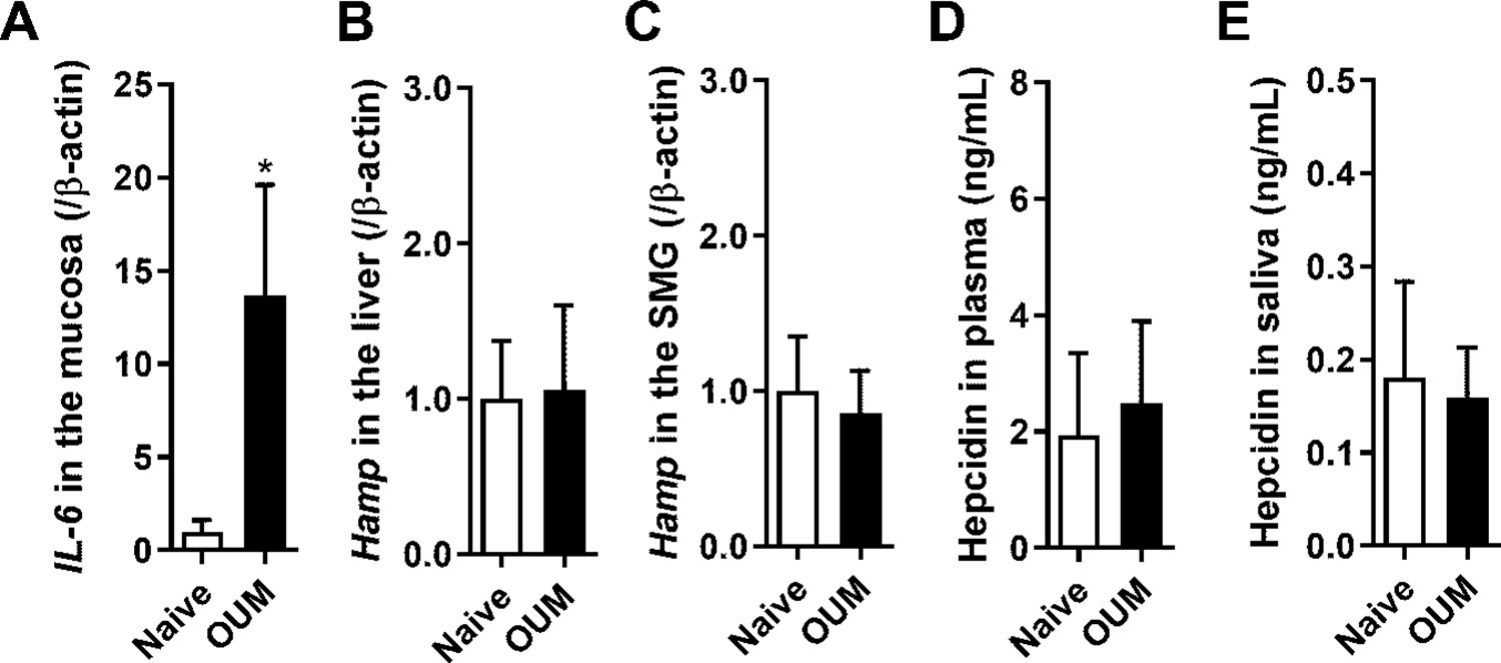
Changes in *IL-6* and *Hamp* mRNA and hepcidin levels in OUM rats. n = 4 in each group. * *p* < 0.05 compared with Naive, Unpaired t-test. **(A)** Relative expression levels of *IL-6* (IL-6 mRNA) in the oral mucosa. **(B, C)** Relative expression levels of *Hamp* in the liver (B) and the submandibular gland (SMG) (C). **(D, E)** Hepcidin levels in plasma (D) and saliva (E) in Naive and OUM.

As IL-6 mediates the amount of hepcidin and consequent hypoferremia during inflammation [32], IL-6 mRNA levels in the ulcer region were investigated in the OUM model. IL-6 mRNA levels were significantly increased in the OUM model compared to naive (Fig 4A, *p* < 0.05), which is consistent with our previous study [14]. Hepcidin mRNA (*Hamp*) levels in the liver, which is the primary source of hepcidin, and the submandibular gland (SMG), were not changed on day 2 after acetic acid treatment (Fig 4B and 4C). Hepcidin protein levels in the plasma and saliva were also unchanged (Fig 4D and 4E).

### Effect of hepcidin on the activity of wide dynamic range (WDR) neurons in the Vi/Vc

**Fig 5:**
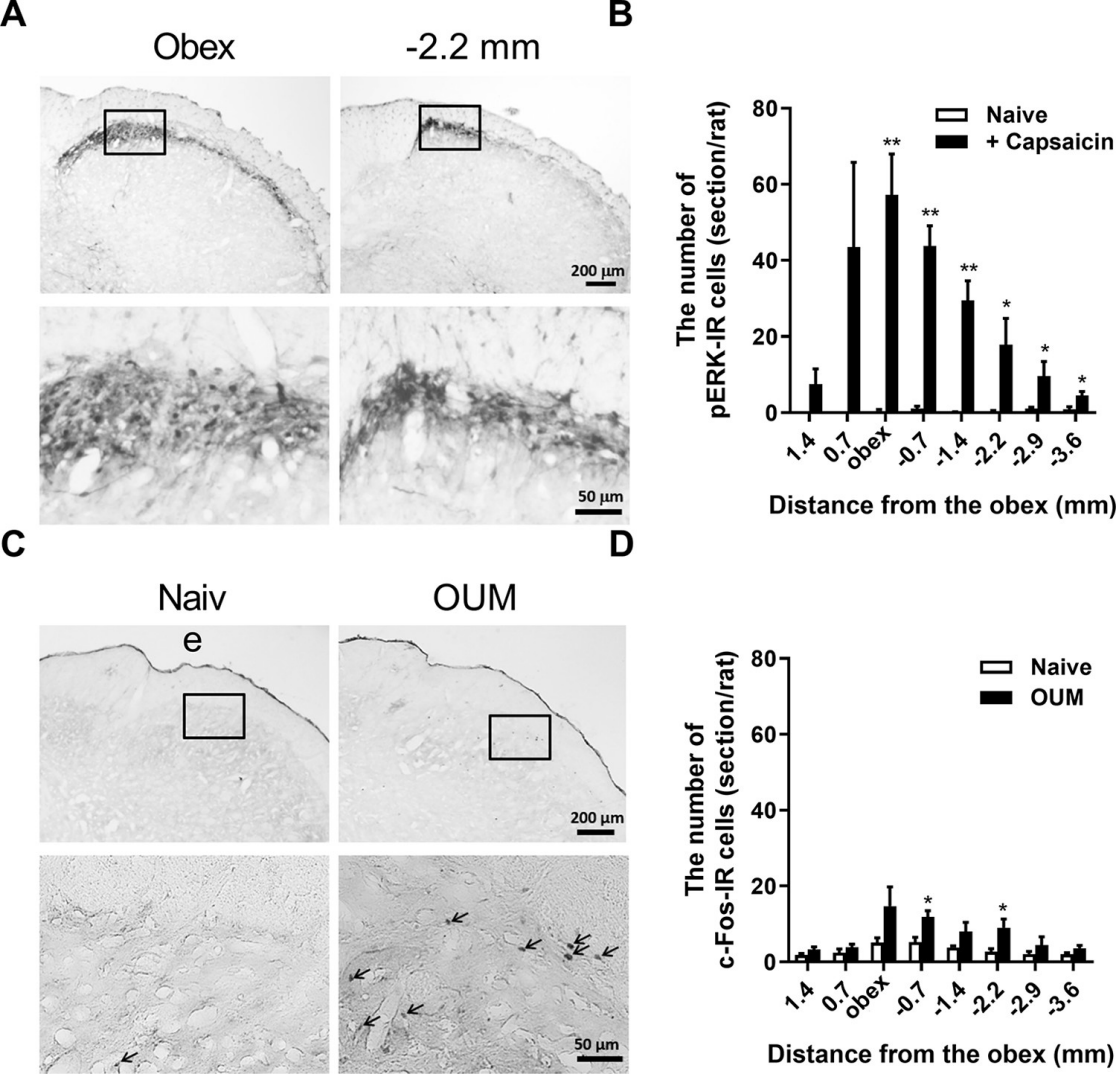
Expression of pERK1/2- and c-Fos-immunoreactive (IR) cells in trigeminal spinal subnucleus interpolaris/caudalis (Vi/Vc). **(A)** Representative photomicrographs of pERK1/2-IR cells in Vi/Vc at obex and -2.2 mm from obex following capsaicin application for 1 min to the oral mucosa in labial fornix region of the inferior incisors. Scale bar, 200 μm (Upper) and 50 μm (Lower). Lower microphotographs are expanded views of the upper squares. **(B)** Rostro-caudal distribution of pERK1/2-IR cells in ipsilateral Vi/Vc following capsaicin application. * *p* < 0.05, ** *p* < 0.01 vs. Naive, Unpaired *t*-test (n = 4–5 in each group). **(C)** Representative photomicrographs of c-Fos-IR cells in Vi/Vc in Naive and OUM model on day 2 after acetic acid treatment in the oral mucosa labial fornix inferior region incisors. Scale bar, 200 μm (Upper) and 50 μm (Lower). Lower microphotographs are expanded views of the upper squares. **(D)** Rostro-caudal distribution of c-Fos-IR cells in ipsilateral Vi/Vc in Naive and OUM model. * *p* < 0.05 vs. Naive, Unpaired *t*-test (n = 5 in each group).

**Fig 6:**
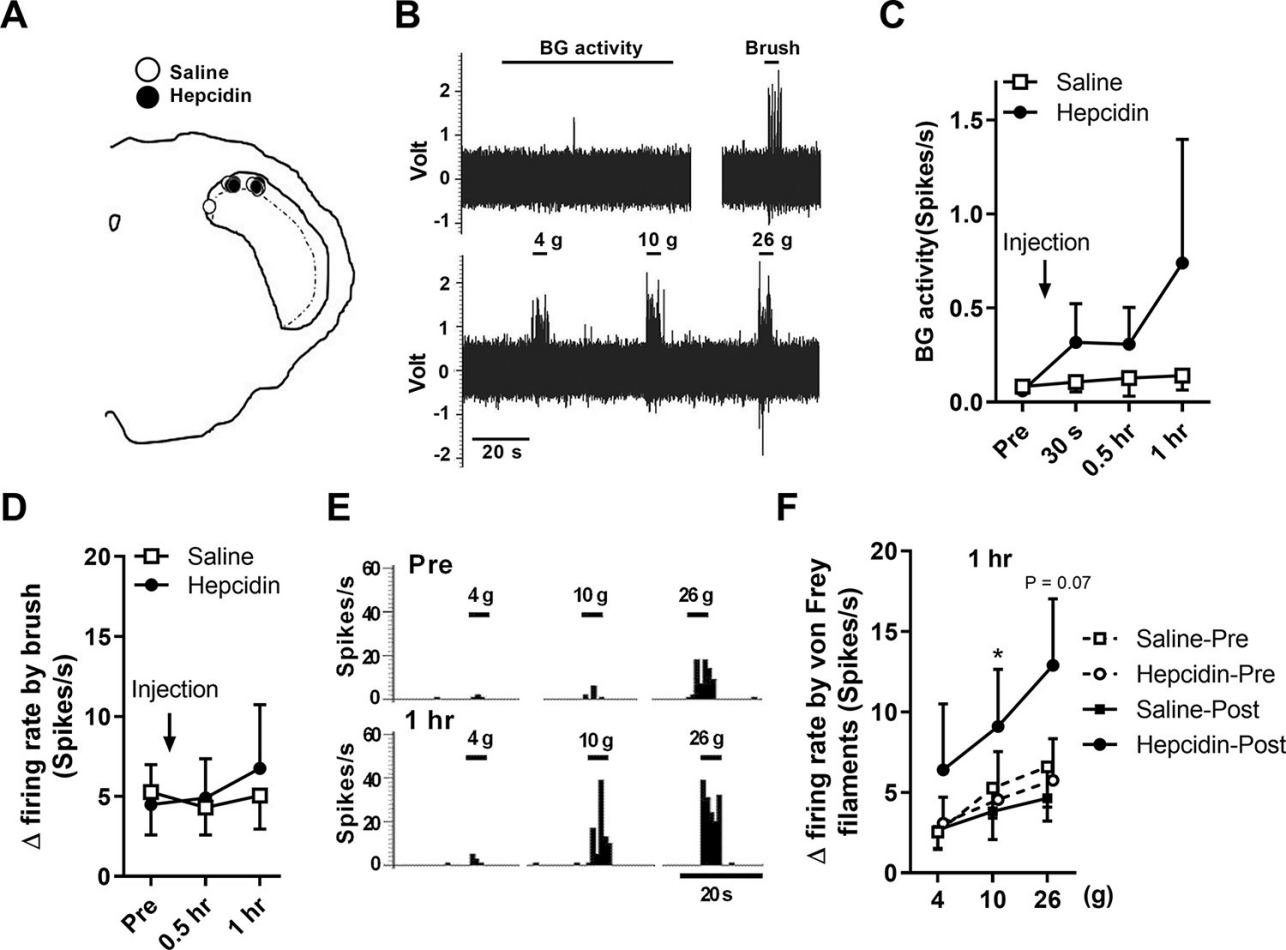
The effects of hepcidin on responses of wide dynamic range (WDR) neurons innervated the oral mucosa in the labial fornix region of the inferior incisors in lamina Ⅰ of the trigeminal spinal subnucleus interpolaris/caudalis (Vi/Vc). Error bars indicate SEM. **(A)** Recording sites are located at obex in Vi/Vc. Open circle: Saline, Closed circle: Hepcidin. **(B)** The raw traces of WDR neurons responses and the frequency of WDR neuronal spikes in response to mechanical stimuli by brush and von Frey filament in naive rats. **(C)** Mean background (BG) activity of WDR neurons for 60 s before and 30 s, 0.5, and 1 hour after saline or hepcidin injections to the oral mucosa (n = 5 in each group). **(D)** Mean *Δ* firing rate of WDR neurons in response to mechanical stimulation using a brush to the oral mucosal region before (pre) 0.5 and 1 hour after saline or hepcidin injections to the oral mucosa (n = 5 in each group). **(E)** Representative example of neuronal activity in response to mechanical stimulation using von Frey filaments to the oral mucosal region before (pre) and 1 hour after saline or hepcidin injections to the oral mucosa. **(F)** Mean *Δ* firing rate of WDR neurons in response to mechanical stimulation using von Frey filaments to the oral mucosal region 1 hour after saline or hepcidin injections to the oral mucosa (n = 5 cells in each group). * *p* < 0.05 vs. hepcidin pre, Paired t-test.

To determine the recording site to respond to the oral mucosa, expression of the pERK1/2 and c-Fos, as markers of neuronal activation, was analyzed following capsaicin application to the oral mucosa or OUM model, respectively. pERK1/2-immunoreactive cells were observed in the dorsal region of the Vi/Vc zone through to the Vc zone (Fig 5A and 5B, *p* < 0.01 at obex, -0.7 and -1.4, *p* < 0.05 at -2.2, -2.9 and -3.6). c-Fos-immunoreactive cells were observed in the same region in the OUM model (Fig 5C and 5D, *p* < 0.05 at -0.7 and -2.2). According to the results, 10 nociceptive neurons were recorded in the Vi/Vc. The neurons were classified as WDR neurons according to their electrophysiological properties in response to mechanical stimulation of the oral mucosa (saline, five neurons; hepcidin, five neurons). The recording site in Vi/Vc neurons is shown in Fig 6A. Fig 6B shows representative examples of neuronal activity (BG activity, typical brush- and von Frey filament-induced responses) recorded from Vi/Vc WDR neurons in naive rats. Hepcidin administration did not alter BG activity and firing rate of WDR neurons in response to mechanical stimulation with a brush (Fig 6C and 6D, respectively). Fig 6E shows typical peristimulus time histograms of Vi/Vc WDR neurons to mechanical stimulation using von Frey filaments to the oral mucosa following hepcidin and saline administration. The firing rates by mechanical stimulation with 15 and 26 g filament 1 h after hepcidin administration (Hepcidin-Post) were higher than that of pre (Hepcidin-Pre) (Fig 6F, *p* < 0.05 between Hepcidin-Pre and Hepcidin-Post at 15 g, *p* = 0.073 between Hepcidin-Pre and Hepcidin-Post at 26 g).

## Discussion

The present study demonstrated that acetic acid-induced OUM elicited neuroplastic changes in various genes in the TG, with an upregulation of 26 genes and a downregulation of 6 genes. In the TG and ulcer regions of the OUM model, there was an increase in the most upregulated gene, *Hamp*, which encodes hepcidin. Our previous study showed that AB inhibited these increases, suppressing bacterial loading and spontaneous and mechanical pain in this model [14]. In this model, hepcidin levels also increased in the ulcer region. IL-6 mRNA, known to regulate hepcidin expression directly [33], was also increased in the ulcer region of the OUM model. In contrast, *Hamp* in the liver, SMG, and hepcidin levels in the plasma and saliva did not change. Hepcidin injection into the oral mucosa enhanced the firing rates of Vi/Vc neurons innervating the oral mucosa in response to mechanical stimulation of the oral mucosa.

On day 2, the acetic acid-treated oral mucosal area demonstrated clear oral ulceration and increased PGE_2_ and bacterial loading, consistent with our previous reports [13–15]. Indomethacin administration and AB treatment inhibited the increased PGE_2_ and bacterial loading, respectively [14]. The spontaneous rubbing time was prolonged, and the mechanical threshold in the oral mucosa was decreased, indicating that spontaneous pain and mechanical allodynia were induced on day 2 after acetic acid treatment in the OUM model. Spontaneous pain depends on COX and is caused by TRPA1 sensitization/activation via the PGE_2_ receptor EP-1 activation and 15d-PGJ2 [10, 15]. Mechanical allodynia is COX-independent and is induced by activation and sensitization of TRP channels, TRPA1 activation by lipopolysaccharide (LPS) in the oral mucosa, endothelin-1-induced TRPV1 sensitization, and TRPV4 sensitization through protease-activated receptor 2 activation by neutrophil elastase [10, 15]. These results imply that OUM is an infectious inflammation. In this gene ontology analysis, upregulated genes in the TG of the OUM model were associated with functions of “Defense response,” “Response to wounding,” and “Inflammatory response.” These functions matched with OUM characterization, indicating changes in OUM-specific genes in the TG.

Hepcidin is an anti-microbial peptide hormone (included in the defensin group) that regulates iron metabolism [33–35]. Most organisms require iron to survive, including oxygen transport by hemoglobin, cellular respiration, DNA synthesis, and reactive oxygen species (ROS) generation for host defense [35, 36]. Hepcidin is produced in hepatocytes with an increase in plasma iron concentration. It binds to an iron exporter, ferroportin (FPN), which is present on the surface of macrophages, endothelial cells, and neurons, decreasing the export of cellular iron via the degradation and internalization of FPN [32, 33]. Hepcidin is also expressed in the brain and regulates iron homeostasis [33]. During inflammation, an inflammatory cytokine IL-6 increases via the LPS/toll-like receptor 4 (TLR4) pathway and upregulates hepcidin expression in macrophages, neurons, and astrocytes via signal transducer and activator of transcription 3 (STAT3) phosphorylation [33, 37, 38].

It has also been reported that the hepcidin mRNA *Hamp* increases in DRGs after peripheral nerve injury and promotes axon regeneration [39]. In this study, *Hamp* in the TG and oral mucosa was upregulated on day 2 after acetic acid treatment in the oral mucosa. In OUM model rats, bacterial loading into the ulcer region caused leukocyte infiltration and increased myeloperoxidase and IL-6 in the ulcer region, leading to spontaneous and mechanical allodynia [14]. Since iron is necessary for bacterial survival, *Hamp/*hepcidin is likely to be upregulated in the ulcer region for anti-microbial action via a decrease in cellular iron export because of FPN degradation. Additionally, IL-6 mRNA was upregulated in the oral ulcer region, suggesting increased *Hamp* and hepcidin levels via the IL-6/STAT3 pathway owing to inflammation in the OUM model. *Hamp* and hepcidin levels in the liver, SMG, plasma, and saliva did not differ between the naive and OUM models. These results show that *Hamp*/hepcidin is produced locally, but not systemically, in the OUM model. Upregulation of *Hamp* in the TG was observed in periodontitis and nerve injury models and the OUM model. Since IL-6 is upregulated in the TG following chronic constriction injury of the infraorbital nerve [40], inflammation and peripheral nerve injury induce *Hamp* upregulation in the TG, possibly via the IL-6/STAT3 pathway.

In this study, the firing rates of Vi/Vc WDR neurons innervating the oral mucosa in response to noxious mechanical stimuli using von Frey filaments (15 and 26 g) were increased 1 h after hepcidin administration into the oral mucosa. These results show that hepcidin enhances noxious responses in Vi/Vc WDR neurons. Overloading of hepcidin causes intracellular iron accumulation via FPN downregulation [33]. The accumulated iron, especially ferrous iron (Fe^2+^), reacts with hydrogen peroxide, which is generated from superoxide radicals under physiological conditions or catalyzed by SOD, resulting in the generation of highly active hydroxyl radicals (∙OH), known as the Fenton reaction [41, 42]. An intrathecal application of the hydroxyl radical donor t-BOOH increased the evoked responses of WDR neurons to mechanical stimulation to the receptive fields starting at 30 min and peaking at 60 min after the application [43]. Furthermore, intrathecal administration of t-BOOH decreased the mechanical threshold of the hind paws 15 min after administration [43]. These reports show that this study may involve hydroxyl radical generation of iron accumulation by hepcidin in the oral mucosa and hepcidin administration-induced enhancement of WDR neuronal responses to mechanical stimulation. Recently, intracellular iron accumulation-induced generation of reactive oxygen species (ROS), including hydroxyl radicals, caused lipid peroxidation, triggering ferroptosis, a type of cell death [42, 44, 45]. Furthermore, the ferroptosis inhibitor liproxstatin-1, which lowers iron levels and lipid peroxidation, alleviates neuropathic pain in a rat model of nerve injury [46]. Taken together, intracellular iron accumulation caused by hepcidin overloading is likely involved in mechanical pain hypersensitivity via neuronal hyperexcitation.

In the present study, the mechanisms how hepcidin is involved in the signaling pathway of OUM-induced pain are not examined. Recently, several drugs for inhibition of hepcidin expression have been identified, such as the hepcidin receptor FPN antagonist and inhibitors of IL-6 and STAT3, which are upstream signaling molecules of hepcidin synthesis [47]. In a future study, we will further investigate the involvement of hepcidin in OUM-induced pain signaling pathways in detail using these drugs.

*Reg3b* is a pancreatitis-associated protein-1 (PAP-1)-coding gene [48]. PAP-1 is associated with regeneration and is upregulated in DRG neurons following inflammation and nerve injury [17]. Furthermore, a recent study has reported that PAP-1 contributes to the maintenance of neuropathic pain via microglial activation [49]. The other upregulated gene in the OUM model, *Serpina3n*, encodes the serine protease inhibitor SerpinA3N, which is expressed in the brain and is associated with apoptosis and wound healing [50]. SerpinA3N has been identified as a pain-inhibiting factor in neuropathic pain models because it suppresses leukocyte elastase activity [51]. In this study, upregulation of these genes was observed in the TG in the OUM model. AB inhibited this up-regulation. Thus, *Reg3b* and *SerpinA3N* are likely to be upregulated in response to infectious inflammation.

In conclusion, various inflammation-related genes in TG were modulated in the OUM model, which was induced by bacterial loading through the ulcer region. In particular, upregulation of *Hamp*/hepcidin in the TG and ulcer regions presumably contributes to iron homeostasis for anti-microbial effects and OUM-induced pain. These findings have significant implications for understanding the molecular mechanisms of OUM-induced pain and will contribute to developing drug therapies for patients with orofacial pain.

## Supporting information

S1 Fig Fold changes in the expression of known pain-related genes in the trigeminal ganglion. **(A)** The expression of upregulated genes in the trigeminal ganglion. **(B)** The expression of downregulated genes in the trigeminal ganglion. Bars represent the fold change in expression in acetic acid-treated rats (n = 5) compared to that in naive rats (n = 5). * *p* < 0.05 and ** *p* < 0.01, Welch’s *t*-test.
(TIF)

S2 Fig Relative expression levels of *Hamp*, *Reg3b*, and *Serpina3n* in dorsal root ganglion (DRG). All bars represent mean ± SEM. **(A)** Summary data from a meta-analysis of published pain microarray studies (GDS2438). Expression of *Hamp* in L5 DRG following spinal nerve ligation (SNL) and L4 SNL (n = 4 in each group). *p < 0.05, compared with sham, Dunnett’s post-hoc test following one-way ANOVA. **(B-D)** Summary data from the meta-analysis of published pain microarray studies (GDS2664 and GDS4625). Expression of *Reg3b* in the DRG in partial nerve ligation (PNL) and CCI models **(B)** and in spinal nerve injury (SNI), spinal nerve ligation (SNL), and CCI models **(C)** (n = 3–9 in each group). Expression of *Serpina3n* in the DRG in SNI, SNL, and CCI models **(D)** (n = 3–9). *p < 0.05, ** *p* < 0.01 compared with sham, Dunnett’s post hoc test following one-way ANOVA.
(TIF)

S1 Raw data (TIF)

## Abbreviations

OUM: oral ulcerative mucositis
PGE2: prostaglandin E2
TG: trigeminal ganglion
DRG: dorsal root ganglion
Reg3b: regulation of islet-derived 3 beta
Ccl2: chemokine [C-C motif] ligand 2
Serpinb1b: serine proteinase inhibitor, clade B,member 1b
Vi/Vc: spinal trigeminal subnucleus interpolaris/caudalis
i.p: intraperitoneally
PB: phosphate buffer
PBS: phosphate buffer saline
IAN-X: inferior alveolar nerve transection
AB: anti-bacterial pretreatments
GO: gene ontology GO
qRT-PCR: quantitative real-time polymerase chain reaction
TBST: Tris-buffered saline containing 0.1% Tween-20
HRP: horseradish peroxidase
TB: Tris buffer
pERK1/2: phosphorylated extracellular signal-regulated kinase
BG: background
mRF: mechanoreceptive field
ANOVA: analysis of variance
CFUs: colony-forming units
GEO: Gene Expression Omnibus
SMG: submandibular gland
LPS: lipopolysaccharide
ROS: reactive oxygen species
FPN: ferroportin
TLR4: toll-like receptor 4
STAT3: signal transducer and activator of transcription 3
PAP-1: pancreatitis-associated protein-1
