# Supplementary figures and images for: Hepcidin expression in the trigeminal ganglion and the oral mucosa in an oral ulcerative mucositis rat model
Source: PLoS One. 2023 Apr 20;18(4):e0284617. doi: 10.1371/journal.pone.0284617 (PMC10118189; doi:10.1371/journal.pone.0284617)

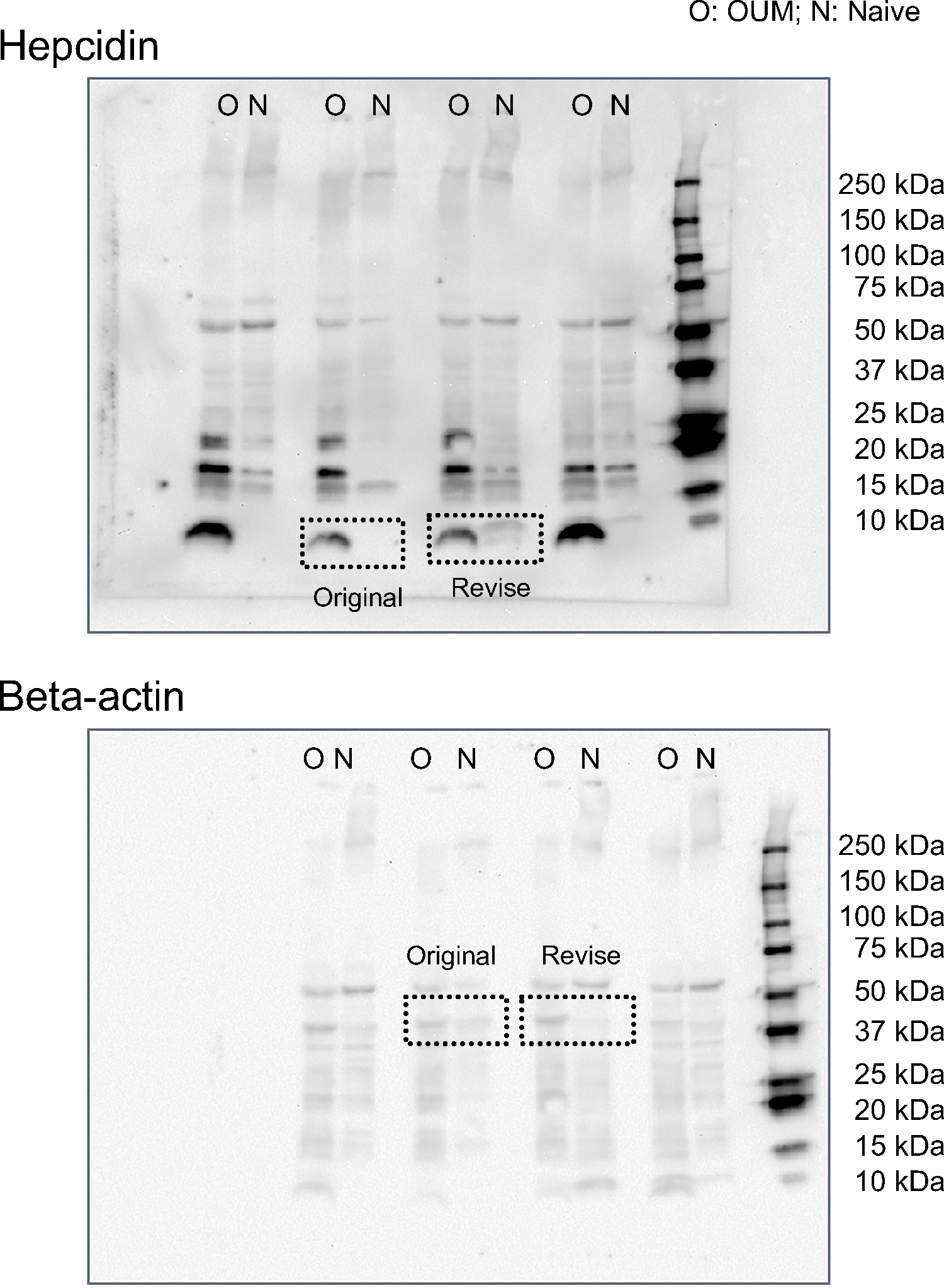

Supplement: S1 Raw data — (TIF) [file pone.0284617.s003.tif]
